# The Effect of Acupuncture on Modulating Inflammatory Cytokines in Rodent Animal Models of Respiratory Disease: A Systematic Review and Meta-Analysis

**DOI:** 10.3389/fimmu.2022.878463

**Published:** 2022-06-15

**Authors:** Serin Lee, Seung-Nam Kim

**Affiliations:** College of Korean Medicine, Dongguk University, Goyang, South Korea

**Keywords:** respiratory diseases, animal models, inflammatory cytokines, acupuncture, meta-analysis

## Abstract

**Purpose:**

Although respiratory diseases (RD) are rapidly becoming a global health issue due to their high mortality and prevalence, there are limitations to the currently available treatments. Acupuncture has been recognized to mitigate many diseases by reducing inflammation and modulating cytokines. However, no systematic analysis has been performed to examine the effects of acupuncture on RD. We aimed to evaluate the effects of acupuncture on rodent animal models of RD.

**Methods:**

PubMed, EMBASE, MEDLINE, and the Research Information Service System were searched to retrieve studies that met our inclusion/exclusion criteria. The quality of each included study was evaluated using a 10-item checklist modified from the Collaborative Approach to Meta-Analysis and Review of Animal Data from Experimental Studies. With adequate data extracted, meta-analysis was performed using RevMan software.

**Results:**

A total of 18 studies were included, and the mean quality assessment was 5.7. The meta-analysis revealed that acupuncture had a significant effect on changing the cytokine levels, including pro-/anti-inflammatory, Th1-, Th2- and Th17- specific cytokines.

**Conclusion:**

Although there were limitations in the number of included studies, the results suggest that acupuncture can be a possible treatment for RD through its modulation of various cytokines, leading to reduced inflammation.

## Introduction

Respiratory diseases (RD) are a major global health issue that have been a serious financial burden owing to the difficulty in treatment. RD encompasses various disorders such as sinusitis, asthma, chronic obstructive pulmonary disease (COPD), and even acute lung injury caused by other diseases. Although the lesions and symptoms of the aforementioned diseases may differ, most of them are associated with acute/chronic inflammation (1). Inflammation is a physiological immune process that includes both inflammatory and restorative responses. Among the factors that participate in this course of action, cytokines function as key mediators with different roles. For example, pro-inflammatory cytokines induce inflammation, while anti-inflammatory cytokines reduce inflammation and participate in the healing process (2). Cytokines can also be categorized according to the type of lymphocytes they are related to. Interleukin (IL)-4, 5, 9, and 13, which are known to be the CD4+ T helper 2 (Th2) cell-responsive cytokines, contribute to allergic airway inflammation. Meanwhile, IL-17, which is released from T helper 17 (Th17) cells, is known to exacerbate chronic lung inflammation (3). Although corticosteroids are currently being used for RD treatment, there are limitations yet to be solved as some cytokines mediate steroid-resistant airway inflammation and obstruction (4).

Acupuncture, which originates from traditional Chinese medicine, has long been acknowledged to mitigate many diseases. Acupuncture at the specific acupoint of ST36 has been reported to promote anti-inflammatory, anti-oxidative, and immune-enhancing effects (5–7). Moreover, the latest studies using animal models of sepsis showed that electroacupuncture at ST36 modulates endotoxin-induced systemic inflammation through the vagal-adrenal anti-inflammatory axis (6, 8). Recently, clinical research in COPD (9), allergic rhinitis (10), asthma (11, 12) showed the possibility of acupuncture as a treatment for these pulmonary diseases. Moreover, there has been increasing evidence of potential therapeutic effects of acupuncture from research on the coronavirus disease 2019 (13). Although pulmonary dysfunction has various etiologies, inflammation mechanisms occur in the lung tissue and fluid. Therefore, it is important to determine which anti-inflammatory mechanisms are linked to the effect of acupuncture on pulmonary diseases.

In this review, we performed a meta-analysis on inflammatory cytokines in various animal models of RD to determine the effects of acupuncture on RD.

## Material and Methods

### Searching

Reports that examined the effect of acupuncture on inflammatory cytokines in rodent models of RD that were written in English were included in the present study. PubMed, EMBASE, MEDLINE, and Research Information Service System were searched from inception until December 2021 using the following search terms: “mouse (mice)” or “rat (rats)”, and “acupuncture (electroacupuncture)” and “respiratory disease.”

### Inclusion/Exclusion Criteria

Studies were included based on the following criteria: subjects (rodent models of RD), interventions (acupuncture as the main intervention but limited to manual acupuncture and electroacupuncture), and outcomes (the levels of each inflammatory cytokine were the main outcomes to evaluate the efficacy of acupuncture). Lung function test data were included as the subsequent outcomes. Along with those that did not provide access to the full text, studies that were not written in English or focused on unrelated topics were excluded. After screening the full text, studies that did not meet our criteria in the methods and results were also excluded.

### Data Extraction

Two authors (Kim and Lee) independently extracted the data on the publication year, name of the first author, type of rodent RD model, disease/condition, sample size, type of acupuncture, and type of specimen. Along with the mean value of inflammatory cytokines within each group (e.g., control group, disease group, intervention group), standard deviation or standard error of the mean were extracted to determine the effect measures and effect size of acupuncture on RD. Each value from the studies was extracted using the Engauge Digitizer software version 12. We estimated the number of animals in groups by calculating the mean number of animals per study. Lung function data, including the type of lung function test and the result, were collected as a secondary outcome.

### Quality Assessment

The methodological quality of each included study was assessed by two authors (Kim and Lee) using a 10-item checklist modified from the Collaborative Approach to Meta-Analysis and Review of Animal Data from Experimental Studies (CAMARADES) checklist (14, 15): publication in a peer-reviewed journal, statements describing control of temperature, random allocation to treatment or control, blinded building of model, use of animals with hypertension or diabetes, blinded assessment of outcomes, use of anesthetic without marked intrinsic properties, sample size calculation, compliance with animal welfare regulations, and declared any potential conflict of interest. The sum of the quality scores was recorded for each article, with a possible total score of 10 points.

### Statistics

In the present study, the ratios of cytokine levels in the disease and intervention groups to the control group were considered as continuous data. To compare studies that used different units for cytokine levels, the difference between the disease/control ratio and acupuncture/control ratio was calculated. Since all 18 studies set RD as an independent variable and analyzed the correlation with the outcomes, the standardized mean difference (SMD) was estimated based on a fixed-effect model. The meta-analysis was performed on each cytokine subgroup using RevMan version 5.4 (Foundation for Statistical Computing, Vienna, Austria). The confidence interval (CI) was established at 95%, and *a p-value* < 0.05 was considered statistically significant. For the assessment of study heterogeneity, chi-squared and I^2^ statistics were used.

## Results

### Study Inclusion

**Figure 1:**
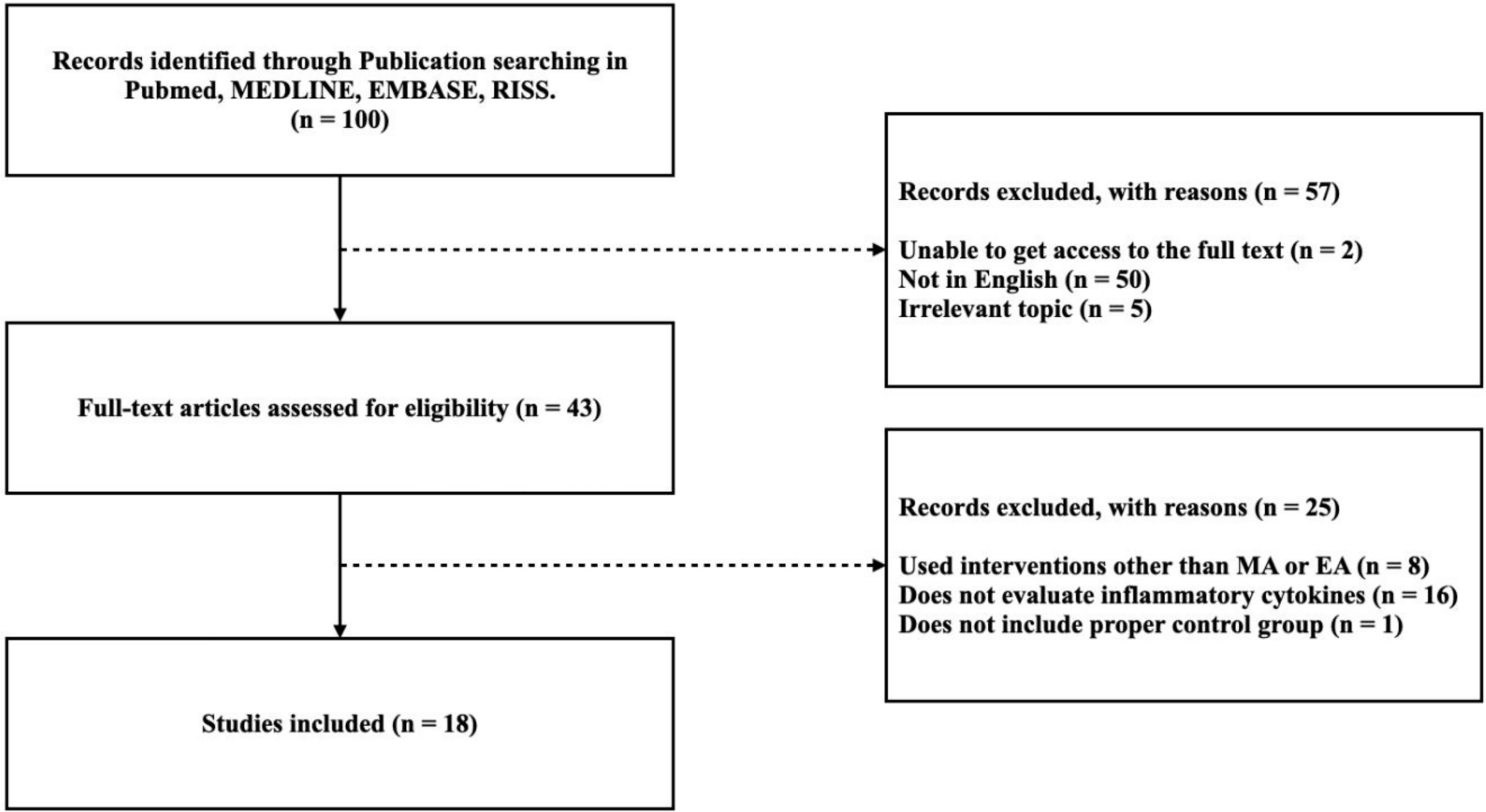
Flowchart of the Study.

Among the 100 studies selected, 57 studies were excluded because 50 were not written in English, five did not cover relevant topics, and two did not allow access to the full text. The full-text screening was performed on the remaining 43 studies, of which 25 were excluded due to deficiencies in the methodology and results. Eight studies used intervention other than manual acupuncture or electroacupuncture, 16 did not evaluate inflammatory cytokines, and 1 study did not include proper control group. Therefore, a total of 18 studies were included in this review. A flow diagram of the study selection process is shown in **Figure 1**.

### Quality Assessment

The quality assessments of the included studies are summarized in **Table 1**. The quality score of the included studies ranged from 3 to 7 out of 10 points: three studies scored 7, eight scored 6, six scored 5, and one scored 3 points. All 18 studies were peer-reviewed and met animal welfare regulations. Twelve studies included statements describing the control of temperature. Fifteen studies specified the random allocation of subjects to treatment or control groups, and six described blinded assessment of the outcomes. Sixteen studies used anesthetics with no intrinsic properties, and 17 declared no potential conflicts of interest with respect to the research. None of the 18 studies conducted blind building of the model or sample size calculation, and none used animals with hypertension or diabetes.

**Table 1:** Quality Assessment.

	Q1	Q2	Q3	Q4	Q5	Q6	Q7	Q8	Q9	Q10	Total
Jiang et al., 2011 (16)	✓						✓		✓		3
Geng et al., 2013 (17)	✓	✓	✓			✓	✓		✓	✓	7
Zhang et al., 2014 (18)	✓	✓				✓	✓		✓	✓	6
Li et al., 2016 (19)	✓	✓	✓				✓		✓	✓	6
Nurwati et al., 2015 (20)	✓	✓	✓						✓	✓	5
Song et al., 2015 (21)	✓		✓				✓		✓	✓	5
Wei et al., 2017 (22)	✓		✓				✓		✓	✓	5
Wang et al., 2017 (23)	✓	✓	✓				✓		✓	✓	6
Dong et al., 2018 (24)	✓	✓	✓				✓		✓	✓	6
Dong et al., 2019 (25)	✓	✓	✓				✓		✓	✓	6
Zhang et al., 2018 (26)	✓	✓	✓				✓		✓	✓	6
Dhar et al., 2019 (27)	✓		✓				✓		✓	✓	5
Huang et al., 2019 (28)	✓	✓	✓			✓	✓		✓	✓	7
Zhou et al., 2019 (29)	✓	✓					✓		✓	✓	5
Cui et al., 2021 (30)	✓	✓	✓			✓	✓		✓	✓	7
Lou et al., 2020 (31)	✓	✓	✓			✓			✓	✓	6
Tang et al., 2021 (32)	✓		✓			✓	✓		✓	✓	6
Zhang et al., 2021 (33)	✓		✓				✓		✓	✓	5

Q1, publication in a peer-reviewed journal; Q2, statements describing control of temperature; Q3, random allocation to treatment or control; Q4, blinded building of model; Q5, use of animals with hypertension or diabetes; Q6, blinded assessment of outcome; Q7, use of anesthetic without marked intrinsic properties; Q8, sample size calculation; Q9, compliance with animal welfare regulations; Q10, declared any potential conflict of interest.

### Study Characteristics

The characteristics of the included studies are summarized in **Table 2**. Among the 18 studies, 10 and eight studies were conducted using rats and mice, respectively. Six studies used an asthma model. Meanwhile, five used a COPD model, and only one used a chronic sinusitis model. Furthermore, six studies used an acute lung inflammation model induced by various preceding conditions, such as amyotrophic lateral sclerosis, thermal injury, cardiopulmonary bypass, or limb ischemia and reperfusion. Eleven studies used electroacupuncture, while seven used manual acupuncture. Specimens were collected to measure the levels of cytokines, including lung tissue, bronchoalveolar lavage fluid, plasma, serum, and nasal tissue.

**Table 2:** Study Characteristics.

	Animal Species	Disease Model	Intervention	Specimen	Cytokines
Jiang et al., 2011 (16)	Mouse	ALS induced lung inflammation	EA	Lung tissue	TNF-ɑ, IL-6
Geng et al., 2013 (17)	Rat	COPD	EA	BALF	TNF-ɑ, IL-1β
Zhang et al., 2014 (18)	Rat	COPD	EA	BALF	TNF-ɑ, IL-1β
Li et al., 2016 (19)	Rat	COPD	MA	BALF	TNF-ɑ, IL-8
Nurwati et al., 2015 (20)	Mouse	Asthma	MA	Plasma	IL-17
Song et al., 2015 (21)	Rat	Thermal injury induced lung inflammation	EA	Plasma	TNF-ɑ, IL-1β, IL-6
Wei et al., 2017 (22)	Mouse	Asthma	MA	Serum	TNF-ɑ, IL-1β, IL-5
Wang et al., 2017 (23)	Rat	CPB induced lung inflammation	EA	Lung tissue	TNF-ɑ, IL-1β
Dong et al., 2018 (24)	Mouse	Asthma	MA	Serum	TNF-ɑ, IL-1β, IL-33
Dong et al., 2019 (25)	Mouse	Asthma	MA	Serum	IL-5, IL-10, IL-13, IL-17A
Zhang et al., 2018 (26)	Rat	COPD	EA	BALF, Lung tissue	TNF-ɑ, IL-6
Dhar et al., 2019 (27)	Rat	CPB induced lung inflammation	EA	Lung tissue	TNF-ɑ, IL-1β, IL-18
Huang et al., 2019 (28)	Rat	CPB induced lung inflammation	EA	BALF, Serum	IL-1β
Zhou et al., 2019 (29)	Mouse	Chronic sinusitis	EA	Nasal tissue	IL-10, IFN-γ
Cui et al., 2021 (30)	Mouse	Asthma	MA	Serum	IL-5, IL-9, IL-13, IL-33, IL-25
Lou et al., 2020 (31)	Rat	Limb ischemia/reperfusion induced lung inflammation	EA	Lung tissue	TNF-ɑ, IL-1, IL-6,
Tang et al., 2021 (32)	Mouse	Asthma	MA	BALF, Serum	IL-4, IL-5, IL-13, IL-17A
Zhang et al., 2021 (33)	Rat	COPD	EA	BALF	TNF-ɑ, IL-10, IL-17

ALS, amyotrophic lateral sclerosis; COPD, chronic obstructive pulmonary disease; CPB, cardiopulmonary bypass; EA, electroacupuncture; MA, manual acupuncture; BALF, bronchoalveolar lavage fluid.

### The Effect of Acupuncture on Inflammatory Cytokines

**Figure 2:**
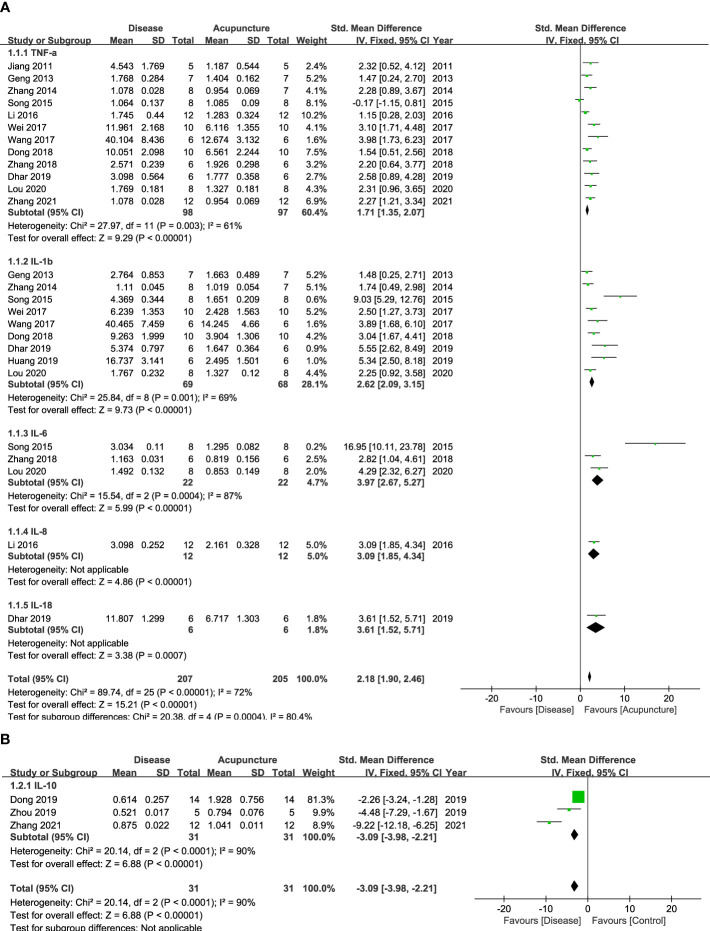
Forest Plot of Pro-/Anti-inflammatory Cytokines. **(A)** Pro-inflammatory Cytokines **(B)** Anti-inflammatory Cytokines.

**Figure 3:**
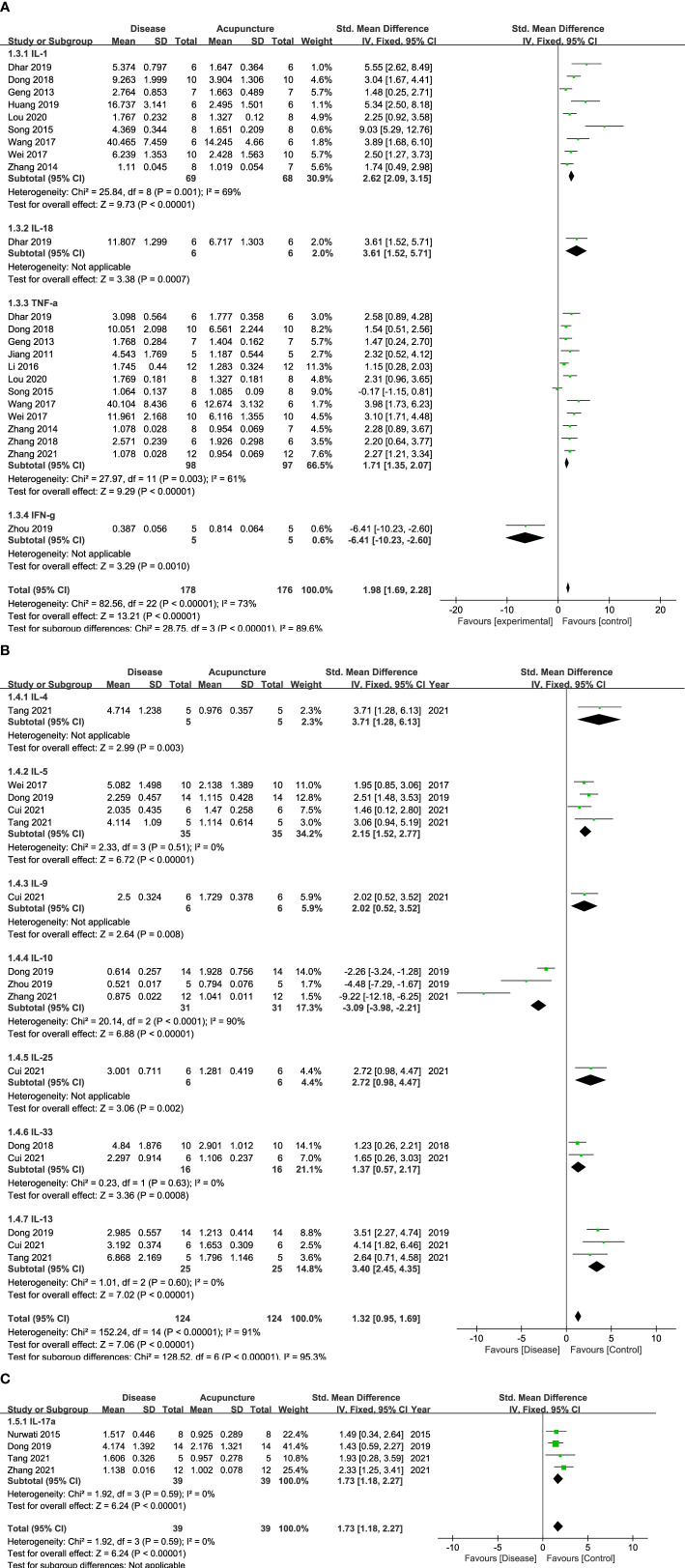
Forest Plot of Th cell-specific Cytokines. **(A)** Th1-specific Cytokines **(B)** Th2-specific Cytokines **(C)** Th17-specific Cytokines.

Cytokines, the principal mediators of inflammation, are composed of various subfamilies that play different roles. First, they are known to have function as pro- or anti-inflammation. Second, cytokines also can be categorized with the immune cells where they are released from, including CD4+ Th1, Th2 or Th17 cells. **Figures 2**, **3** show that 14 cytokines were analyzed in this study. Due to the diverse properties of these cytokines, we performed the meta-analysis by categorizing cytokines into separate subgroups: (1) pro-/anti-inflammatory cytokines, and (2) Th1-, Th2-, and Th17-specific cytokines. The results revealed that rodent animal models with RD had higher levels of pro-inflammatory cytokines than the control group, and acupuncture lowered these levels (n = 207 for disease group, n = 205 for acupuncture group, SMD 2.18 [95% CI 1.90 ~ 2.46]; *p* < 0.00001; heterogeneity *X* ^2^ = 89.74, *I* ^2^ = 72%, **Figure 2A**). Anti-inflammatory cytokines showed the opposite result, showing a decrease in the disease model and an increase with acupuncture treatment (n = 31, SMD -3.09 [95% CI -3.98 ~ -2.21]; *p* < 0.00001; heterogeneity *X* ^2^ = 20.14, *I* ^2^ = 90%, **Figure 2B**). Most Th-1 specific cytokines seemed to have decreased by acupuncture treatment except for IFN-𝛾. (n=178, SMD 1.98 [95% CI 1.69 ~ 2.28]; p < 0.00001; heterogeneity *X* ^2^ = 28.75, *I* ^2^ = 73%, **Figure 3A**). Th2-specific cytokines had a similar tendency to that of pro-inflammatory cytokines (n = 124, SMD 2.24 [95% CI 1.84 – 2.64]; *p* < 0.00001; heterogeneity *X* ^2^ = 15.77, *I* ^2^ = 30%, **Figure 3B**). Lastly, Th-17 specific cytokines, which increased in the disease groups, were also shown to be regulated by acupuncture treatment (n = 39, SMD 1.73 [95% CI 1.18 – 2.27]; *p* < 0.00001; heterogeneity *X* ^2^ = 1.92, *I* ^2^ = 0%, **Figure 3C**).

### The Effect of Acupuncture on the Lung Function in Animal Models of RD

**Table 3** shows how acupuncture affected lung function in RD models after examining the results of pulmonary function tests (PFTS), lung resistance (RL), and lung dynamic compliance (Cdyn). PFTS data included forced expiratory volume in 0.1s (FEV_0.1_), forced expiratory volume in 0.3s (FEV_0.3_), the ratio between the aforementioned two and forced vital capacity (FEV_0.1_/FVC, FEV_0.3_/FVC), inspiratory capacity, peak expiratory flow, and minute volume. Among the 18 studies, seven measured RL and Cdyn, three conducted PFTS, and eight did not mention any lung function tests. Acupuncture was found to increase Cdyn and alleviate RL. It was also shown that acupuncture improved lung function in general by increasing FEVs, FVC, inspiratory capacity, peak expiratory flow, and minute volume.

**Table 3:** Results of Lung Function Tests.

	Results	Lung Function Tests
Jiang et al., 2011 (16)	N/A	
Jiang et al., 2011 (16)	Improved	RL ↓, Cdyn ↑
Zhang et al., 2014 (18)	Improved	FEV_0.1_ ↑, FEV_0.3_ ↑, FEV_0.1_/FVC ↑ FEV_0.3_/FVC ↑
Li et al., 2016 (19)	Improved	IC ↑, PEF ↑, MV ↑
Nurwati et al., 2015 (20)	N/A	
Song et al., 2015 (21)	N/A	
Wei et al., 2017 (22)	Improved	RL ↓, Cdyn ↑
Wang et al., 2017 (23)	N/A	
Dong et al., 2018 (24)	Improved	RL ↓, Cdyn ↑
Dong et al., 2019 (25)	Improved	RL ↓, Cdyn ↑
Zhang et al., 2018 (26)	Improved	FEV_0.1_ ↑, FEV_0.3_ ↑, FEV_0.1_/FVC ↑ FEV_0.3_/FVC ↑
Dhar et al., 2019 (27)	N/A	
Huang et al., 2019 (28)	N/A	
Zhou et al., 2019 (29)	N/A	
Cui et al., 2021 (30)	Improved	RL ↓, Cdyn ↑
Lou et al., 2020 (31)	N/A	
Tang et al., 2021 (32)	Improved	RL ↓, Cdyn ↑
Zhang et al., 2021 (33)	Improved	RL ↓, Cdyn ↑

N/A, not applicable; RL, lung resistance; Cdyn, dynamic compliance; FEV0.1, forced expiratory volume in 0.1s; FEV0.3, forced expiratory volume in 0.3s; FVC, Forced vital capacity; IC, inspiratory capacity; PEF, peak expiratory flow; MV, minute volume.

## Discussion

Recently, a number of clinical studies have reported the effect of acupuncture on RD. Acupuncture enhanced the strength of diaphragm while relieving the respiratory muscle fatigue of COPD patients (9), mitigated overall symptoms of allergic rhinitis/rhinoconjunctivitis (10), and showed therapheutic effects on bronchial asthma as well (12). However, no studies oversee the RD animal studies and systematically analyzes the effect of acupuncture on inflammatory cytokine as its underlying mechanism. In this review, we systematically analyzed 18 acupuncture studies that used rodent models of RD to determine whether acupuncture can improve RD symptoms and/or pathology. Rat/mouse models of asthma, COPD, cardiopulmonary bypass-, amyotrophic lateral sclerosis-, reperfusion-induced lung inflammation, and chronic sinusitis were administered with manual or electro-acupuncture, and the lung functions were then examined. To test lung function, the RL, Cdyn, FEV, FVC, peak expiratory flow, and minute volume were tested, and the results showed that acupuncture improved lung function in various inflammatory pulmonary disease animal models.

The following inflammatory cytokines were examined in lung tissue, fluids, or serum/plasma samples from animal models. Inflammatory cytokines can be specifically classified according to their functions: those that promote inflammation are known to be pro-inflammatory, while those that engage in restoring damages are considered anti-inflammatory cytokines. In pulmonary diseases, pro-inflammatory cytokines are known to trigger mucus secretion and airway fibrosis (34), while anti-inflammatory cytokines have been reported to be deficient, especially in asthma and COPD (35). In our results, pro-inflammatory cytokines, such as IL-1, 6, 8, 18, and TNF-α, were lowered by acupuncture. In contrast, anti-inflammatory cytokines, such as IL-10, showed an opposite tendency. Thus, our results suggest that acupuncture can possibly ameliorate inflammation by inducing anti-inflammatory cytokines while reducing pro-inflammatory ones depending on the set condition. Meanwhile, some cytokines are not simple enough to categorize by their pro-/anti-inflammatory roles. IFN-𝛾, for example, is a Th1 cytokine that counterbalances Th2 cytokines. However, its anti-inflammatory action has also been reported, suggesting that the functional roles of some cytokines are complex in immune diseases (36, 37).

Interestingly, acupuncture showed that it could possibility reduce Th2-related cytokine levels in asthma models, which might help maintain the balance between Th1 and Th2 cytokines. Asthma, an allergic inflammation driven by Th2 lymphocytes, shows representative pathology of higher levels of Th2 cytokines compared to Th1 cells (1). According to our study, Th2 cytokines such as IL-4, 5, 9, 13, 25, and 33 were downregulated by acupuncture treatment. This allows an additional interpretation of how acupuncture can normalize Th2 cytokine levels to alleviate inflammation in asthma. Furthermore, IL-17, which is released by Th17 cells, leads to the aggravation of allergic inflammation and airway hyper-responsiveness by causing neutrophilia and eosinophilia (38, 39). In our study, acupuncture significantly modulated abnormal levels of IL-17, again implying its effect on RD *via* Th17 cell control. As Th2 and Th17 cells play a crucial role in RD pathology (40), the T-cell-regulating ability of acupuncture deserves further investigation (41).

There are a few limitations to the present study. First, cytokines were not classified in a disease-specific manner, as this study encompassed overall effects of acupuncture on RD. Second, the fact that cytokines can play a different role depending on the situation could obscure the purpose of this review. This also caused some difficulties in categorizing them. In order to prevent aforementioned problem, we referred to how each study interpreted the role of cytokines. Third, the number of included studies was not conducive for a thorough systematic review, which implies the limited research investigating the effects of acupuncture. However, it is meaningful in that this study evaluated the efficacy of acupuncture on RD for the first time by analyzing inflammatory cytokines. In order to reveal the hidden mechanism of acupuncture, further research investigating each RD is needed.

## Conclusion

In summary, this review showed that acupuncture treatment might ameliorate lung dysfunction in RD by regulating inflammatory cytokines. Results showed that when mitigating RD, acupuncture modulates pro/anti-inflammatory, Th1-, Th2-, and Th17-specific cytokines. We look forward to further research on the therapeutic effects of acupuncture and hope that the current review will be a good reference in this field of study.

## Data Availability Statement

The raw data supporting the conclusions of this article will be made available by the authors, without undue reservation.

## Author Contributions

SL searched the database and extracted data. S-NK designed and supervised the study. SL and S-NK analyzed the data and wrote the paper. All authors contributed to the article and approved the submitted version.

## Funding

This work was supported by the National Research Foundation of Korea funded by the Korean government (MSIT) (NRF-2020R1C1C1004107) and from the Ministry of Health & Welfare through the Korea Health Industry Development Institute (KHIDI) (grant no. HF21C0018).

## Conflict of Interest

The authors declare that the research was conducted in the absence of any commercial or financial relationships that could be construed as a potential conflict of interest.

## Publisher’s Note

All claims expressed in this article are solely those of the authors and do not necessarily represent those of their affiliated organizations, or those of the publisher, the editors and the reviewers. Any product that may be evaluated in this article, or claim that may be made by its manufacturer, is not guaranteed or endorsed by the publisher.
